# Artificial Intelligence and Radiomics: Clinical Applications for Patients with Advanced Melanoma Treated with Immunotherapy

**DOI:** 10.3390/diagnostics13193065

**Published:** 2023-09-27

**Authors:** Jeremy McGale, Jakob Hama, Randy Yeh, Laetitia Vercellino, Roger Sun, Egesta Lopci, Samy Ammari, Laurent Dercle

**Affiliations:** 1 Department of Radiology, New York-Presbyterian Hospital, New York, NY 10032, USA; 2 Queens Hospital Center, Icahn School of Medicine at Mt. Sinai, Queens, NY 10029, USA; 3 Molecular Imaging and Therapy Service, Department of Radiology, Memorial Sloan Kettering Cancer Center, New York, NY 10065, USA; 4 Nuclear Medicine Department, INSERM UMR S942, Hôpital Saint-Louis, Assistance-Publique, Hôpitaux de Paris, Université Paris Cité, 75010 Paris, France; 5 Department of Radiation Oncology, Gustave Roussy, 94800 Villejuif, France; 6 Nuclear Medicine Unit, IRCCS—Humanitas Research Hospital, 20089 Rozzano, MI, Italy; 7 Department of Medical Imaging, BIOMAPS, UMR1281 INSERM, CEA, CNRS, Gustave Roussy, Université Paris-Saclay, 94800 Villejuif, France; 8 ELSAN Department of Radiology, Institut de Cancérologie Paris Nord, 95200 Sarcelles, France

**Keywords:** melanoma, immunotherapy, immune checkpoint inhibitor, radiomics, artificial intelligence, immunoPET, medical imaging, oncology

## Abstract

Immunotherapy has greatly improved the outcomes of patients with metastatic melanoma. However, it has also led to new patterns of response and progression, creating an unmet need for better biomarkers to identify patients likely to achieve a lasting clinical benefit or experience immune-related adverse events. In this study, we performed a focused literature survey covering the application of artificial intelligence (AI; in the form of radiomics, machine learning, and deep learning) to patients diagnosed with melanoma and treated with immunotherapy, reviewing 12 studies relevant to the topic published up to early 2022. The most commonly investigated imaging modality was CT imaging in isolation (*n* = 9, 75.0%), while patient cohorts were most frequently recruited retrospectively and from single institutions (*n* = 7, 58.3%). Most studies concerned the development of AI tools to assist in prognostication (*n* = 5, 41.7%) or the prediction of treatment response (*n* = 6, 50.0%). Validation methods were disparate, with two studies (16.7%) performing no validation and equal numbers using cross-validation (*n* = 3, 25%), a validation set (*n* = 3, 25%), or a test set (*n* = 3, 25%). Only one study used both validation and test sets (*n* = 1, 8.3%). Overall, promising results have been observed for the application of AI to immunotherapy-treated melanoma. Further improvement and eventual integration into clinical practice may be achieved through the implementation of rigorous validation using heterogeneous, prospective patient cohorts.

## 1. Introduction

Melanoma is among the most commonly diagnosed cancers in the United States and is a significant driver of cancer-related deaths worldwide [1]. While early-stage, non-progressive melanoma has a favorable prognosis with good 5-year survival rates after surgical resection (up to 98.4%), there is a precipitous drop-off in survival for more advanced stages (63.6% and 22.5% for regional and metastatic disease, respectively) [2,3]. This dichotomy of outcomes has prompted efforts toward the earlier detection of melanoma, while it is still surgically resectable, and improved treatment response monitoring after the diagnosis of any stage disease. Research on the combination of immunotherapy and artificial intelligence (AI) seeks to address these problems. The former offers an innovative therapeutic strategy for malignant melanoma management, and the latter provides an updated means for both the a priori prediction of treatment responders and the accurate evaluation of efficacy after treatment initiation. In this review, we provide background information on immunotherapy and AI before elaborating on the present landscape of their combination in patients with malignant melanoma. We then highlight some significant recent advances and possible future directions for investigation.

## 2. Development of Immunotherapy

In recent years, the introduction of immunotherapy into clinical practice has dramatically improved treatment outcomes for various malignancies, including lung, head and neck, bladder, and gastrointestinal cancers as well as melanoma and various lymphomas. Immunotherapy is designed to weaponize a patient’s own immune system against cancer, and it was first introduced in the form of a CTLA-4 inhibitor, ipilimumab, indicated for metastatic and unresectable melanoma [4]. Over the decade since its introduction, several additional agents have been developed as standalone, adjuvant, or rescue therapies, including monoclonal antibodies targeting a wide range of immune-related receptors and ligands, as well as oncolytic viruses and immunocytokines [5,6,7,8,9]. These treatments target various biological mechanisms; for example, certain tumors have an evolved ability to “shut down” the native immune response via the binding of host T-cell programmed cell death protein 1 (PD-1), cytotoxic T-lymphocyte associated protein 4 (CTLA-4), or lymphocyte activation gene 3 (LAG-3) receptors. Interactions at these receptors temper immune cell activity, and while this serves a natural purpose of preventing overactivity and auto-immunity in a healthy individual, exploitation by tumor cells facilitates escape from immune surveillance. Immune checkpoint inhibitors make up a class of immunotherapy that specifically targets these receptors, preventing the binding of tumor cells and, in effect, releasing the “brakes” of a patient’s immune system. Oncolytic viruses, on the other hand, use an engineered viral platform (e.g., adenovirus, herpes simplex virus, and poxvirus, among others) to specifically infect cancer cells, leading to their eventual deaths and creating targetable antigens in the process, thus helping a patient build tumor-specific immunity [10,11]. In addition, there is remarkable flexibility in viral genome editing, which can be used to express immune-system-activating factors (e.g., cytokines) from within a tumor, ultimately recruiting host immune cells to the site of malignancy.

## 3. New Patterns of Response and Progression with Immunotherapy

The novelty of immunotherapy, when paired with individual tumor heterogeneity, precipitates many response patterns that are not accurately characterized by conventional size-based imaging criteria, such as the Response Evaluation Criteria in Solid Tumors (RECIST). Among these is pseudoprogression, in which there is a transient increase in tumor size before an eventual response, potentially correlating with either a lag time before immune system activation or local inflammation caused by host immune invasion [5,12]. This is particularly interesting in relation to melanoma, in which one study demonstrated an eventual diagnosis of pseudoprogression in 17.9% of patients allowed to continue treatment after the initial worsening of the disease [13]. Additionally, “mixed” responses indicate tumors with discordant responses to treatment (i.e., some lesions may grow while others shrink), corresponding to the heterogeneous nature of individual tumors and *a priori* immune cell infiltration [14]. There are no specific baseline phenotypes that signal impending atypical responses, yet these responses are critical to identify as soon as they arise; inaccurate response classification may lead to the early termination of treatment if traditional size-based imaging criteria are being followed strictly. Another interesting response pattern involves the abscopal effect, in which the treatment of a single target lesion (via local application of medication or radiation treatment) leads to a decrease in the size of distant tumors within the same patient. This phenomenon is thought to arise from the release of tumor antigens from the primary lesion upon treatment, subsequently activating a patient’s immune system against similar tumors elsewhere in the body.

Perhaps more important still is the possibility for hyperprogression of disease as a result of immunotherapy. This rare response pattern consists of rapid, large-volume tumor growth and dissemination after the initiation of treatment due to a yet unknown mechanism. Identifying hyperprogression at the earliest possible moment is critical, though it is a feat that has been difficult to accomplish and has historically resulted in poor patient outcomes [15].

## 4. Melanoma Response to Immunotherapy

An dramatic improvement in survival has been observed in cases of advanced melanoma following the introduction of immunotherapy. In 2019, Larkin et al. reported a five-year overall survival (OS) rate of 52% for patients treated with nivolumab and ipilimumab [16]. These rates were calculated based on patients in the CheckMate 067 trial, and when the same patients were analyzed six and a half years later, the OS remained high at 49% [17]. A comparison of these figures to a 2011 report by Garbe et al., in which 5-year OS was less than 10% for patients with advanced melanoma, highlights the impact that immunotherapy has had in this field [18].

Melanoma’s particular susceptibility to immunotherapy is most likely due to its high immunogenicity, meaning that the cancer itself is distinctly immune-provoking and vulnerable to breakdown by a patient’s innate defenses [19]. This is evident due to the prevalence of melanoma in immune-compromised patients, the occurrence of disseminated disease with an absent primary lesion indicating spontaneous regression, and the documented high tumor mutational burden of many melanomas [20]. Additionally, both primary and metastatic melanoma lesions have been found to permit rapid reactive lymphocyte infiltration, indicating that they are somewhat less likely to cordon themselves off in defense against the host immune system. Despite these immunogenic factors, melanoma remains highly lethal due to its ability to evolve and disseminate quickly in the face of selective environmental pressures. In this scenario, immunotherapy is particularly effective, as the host immune system is already functioning properly; however, it is not strong enough to offset the disease’s evasive tactics. By amplifying the immune response—activating immune cells already within a tumor, providing additional antigens for further tumor recognition, or overexpressing inflammatory cytokines to recruit additional defenders—the balance is tipped towards regression and remission.

However, though immunotherapy has demonstrated impressive efficacy in improving OS, it is estimated that 40% to 65% of patients demonstrate minimal or no response at the beginning of treatment, which has been termed primary resistance [21]. The mechanism of treatment resistance is not currently known but is theorized to result from insufficient antigen presentation, host T-cell activation, or immune infiltration of tumor microenvironments. Early identification of these patients, allowing for a switch to other therapeutic strategies, is key to improving overall outcomes.

## 5. Immune-Related Adverse Events

Immune-related adverse events (irAEs) are side effects specific to immunotherapy that result from treatment-induced inflammation resulting in collateral damage to non-malignant patient tissue [22]. It can be difficult to both identify irAEs outright and differentiate them from progression due to the similarity of imaging features between errant inflammation of host tissues and intended immune cell activation against a tumor. However, this task remains crucial because of the potential for intolerable morbidity that may delay or disrupt treatment courses. Effects can appear in any organ system, with severity ranging from mild (requiring treatment with corticosteroids) to life-threatening, depending on the specific drug, dosing regimen, and individual patient characteristics such as preexisting autoimmune disease, hereditary genetic polymorphisms, or even smoking status, among others [23,24,25]. The CheckMate 067 trial found that 36% of metastatic melanoma patients receiving combination therapy of nivolumab plus ipilimumab experienced serious irAEs, primarily localizing in the skin (pruritis, rash, and vitiligo), gastrointestinal tract (diarrhea and colitis), liver (transaminitis and hepatitis), and thyroid (hypo-/hyperthyroidism and thyroiditis), with an even further increased incidence in the 4-year follow up report [26]. Other studies have suggested that, overall, up to 92% of patients experience some type of irAE, regardless of the treatment regimen employed [12]. Vitiligo is one of the most commonly encountered irAE observed in the treatment of melanoma and is interestingly associated with better OS, likely due to its indication of strong host immune system activation and the relatively low morbidity of the effect [27]. Generally, however, irAEs pose a risk to patient safety, with higher risks observed in combination drug regimens (e.g., nivolumab and ipilimumab together), and must be actively monitored throughout treatment [28].

## 6. Intratumoral Immunotherapy

To potentially mitigate systemic inflammatory effects, several groups are currently investigating locally administered intratumoral immunotherapy [29]. This strategy spares patients from the irAEs of whole-body immunotherapy and takes advantage of the abscopal effect described vide supra. Better avoidance of irAEs could allow for the greater deployment of combination therapies, including the recently published relatlimab–nivolumab combination, which has been shown to provide greater progression-free survival than monotherapy, as well as a separate technique involving the concomitant application of intratumoral oncolytic peptides and viruses with immune checkpoint inhibitors [30,31,32,33]. However, the assessment and prediction of responses to intratumoral treatment have yet to be widely investigated and may require additional tools for accurate evaluation.

## 7. AI and Radiomics: Concept

Predicting and tracking the labile effects of immunotherapy is an area of active research, driven in large part by advances in medical imaging. While computed tomography (CT), positron emission tomography (PET), and magnetic resonance imaging (MRI) are already widely employed for tumor characterization, staging, and response assessment, these methods must be augmented to approach the novel responses described above.

A potential method for the improvement of imaging-based monitoring is the implementation of AI, in the form of radiomics and deep learning, that allows for the extraction and analysis of imaging features not typically interpretable by radiologists [34]. At a high level, the process is relatively simple: imaging features are created by describing standardized relationships between pixels or voxels (the three-dimensional equivalent of a pixel) and their individual intensities within a medical image. These features are subsequently combined in multiple regression models used for the classification or prediction of specific clinical endpoints and are stress-tested on external datasets in order to define model performance and generalizability. The central theory is that an amalgamation of radiomics features with certain assigned weights creates a tumor “imaging phenotype”. This phenotype can either be correlated with outcome (e.g., overall/progression-free survival or durable clinical benefit) or, given enough data, used by clinicians for decision making regarding a particular course of treatment. For example, a certain imaging phenotype may be associated with higher tumor PD-L1 expression, thus indicating a cancer that is more susceptible to anti-PD-L1 monoclonal antibody therapy. Additionally, the phenotype may indicate a tumor with a high level of infiltrating CD4 lymphocytes, implying that immune activation via immunotherapy would be beneficial given that the host immune system has not yet been “locked out” of the malignant space. These immune-rich presentations have been associated with better outcomes, while an “immune-desert” phenotype, describing a paucity of host immune infiltration, indicates a low potential for response [35]. There is also an “excluded” phenotype, in which cancer cells secrete factors (e.g., TGF-β) that invoke stromal and extracellular matrix proliferation around a tumor, effectively creating a physical barrier against host immune cells [36].

Under the broader umbrella of AI, radiomics involves the hand selection of individual imaging features based on previously defined associations with biological phenomena (e.g., heterogeneity of tumors on imaging could relay information about vascularization, inflammation, or necrosis within a tumor). On the other hand, deep learning requires much larger datasets and involves the autonomous selection of features that an algorithm, or convolutional neural network (CNN), determines to be most predictive of an outcome. While the latter may utilize a more intangible and, therefore, less accessible method of model generation, it harbors potential to describe relationships and imaging patterns that may be missed, even by subject matter experts.

While AI approaches aim to address the problems associated with qualitative imaging analysis, many challenges must first be overcome before widespread clinical application. Briefly, the development of accurate predictive models relies on large, high-quality datasets that can be used for training and validation. These datasets, as will be discussed vide infra, should be diverse and spread across institutions to generate the most heterogeneous and broadly representative patient sample possible. In recruiting large cohorts, another challenge arises regarding the standardization of imaging techniques between patients and institutions such that images can be more easily collated and reliably used without interference from variable acquisition processes. Lastly, while predictive models built using various techniques, analytical methods, and features create broad variability in what is available for use in the field, there exists an ongoing issue of reproducibility. Moving forward, there is a need to more thoroughly define methods and best practices to allow for better comparison of studies.

## 8. AI and Radiomics: Current Landscape in Relation to Melanoma Imaging

A recent review surveyed studies published up to early 2022 that applied AI and radiomics to patients treated with immunotherapy [37]. Articles were included if they involved the application of immunotherapy to patients with cancer (or in murine models of human cancers) and if they employed AI (including radiomics) in combination with PET, CT, or MRI imaging. Of the 87 articles in the final analysis, 12 (13.7%) included patients with melanoma, making it the second-most represented primary disease behind non-small cell lung cancer [38,39,40,41,42,43,44,45,46,47,48,49].

**Figure 1:**
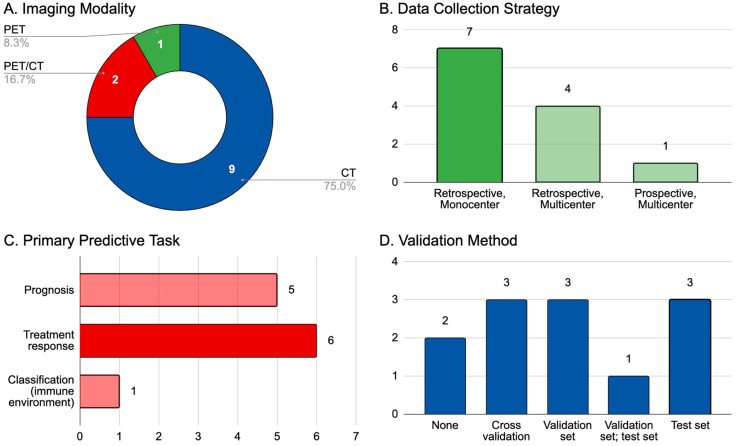
Summary of data extracted from the 12 relevant studies identified in an early 2022 review of the field. (**A**) primary imaging modality employed (PET indicates ^18^F-FDG-PET imaging); (**B**) data collection strategy classified by two variables: time course (retrospective or prospective) and center involvement in patient recruitment (single or multiple institutions); (**C**) primary predictive aim of the AI model built in the study, e.g., prognosis (measures of overall or progression-free survival and durable clinical benefit), treatment response (predictions of clinical endpoints as defined by RECIST 1.1 or similar criteria), classification (description of immune environment, namely, tumoral infiltration of CD8+ T-cells); (**D**) validation method used to test the reported AI model.

A summary of these studies is displayed in Figure 1. Three of the twelve studies (25%) included melanoma patients in mixed-cancer investigations, and the median [interquartile range] melanoma-specific cohort size was 56 [39]. Most patients were evaluated retrospectively (*n* = 11, 91.7%), from either single (*n* = 7, 59.3%) or multiple (*n* = 5, 41.7%) institutions, and the most commonly studied imaging modalities were CT (*n* = 9, 75%), PET/CT (*n* = 2, 16.7%), or PET alone (*n* = 1, 8.3%). While MRI was used in 14 out of the 87 overall papers (16.1%), it was not employed in studies with melanoma patients.

## 9. AI in Radiomics: Predictive Aims

In the 2022 review, AI-based predictive models were built for several different primary tasks within the sphere of immunotherapy-treated melanoma. Primarily, investigators sought to predict treatment response (*n* = 6, 50%), utilizing RECIST 1.1 or similar criteria to define primary disease endpoints. Almost an equal number of studies (*n* = 5, 41.7%) used AI to make predictions on measures of prognosis, including overall or progression-free survival (the interval between treatment initiation and the first evidence of clinical or radiological disease progression) as well as durable clinical benefit (progression-free survival past a certain time point). The last study (*n* = 1, 8.3%) involved using AI to describe host CD8+ T-cell infiltration of malignant lesions. Interestingly, no melanoma studies explored tumor phenotyping, such as predicting PD-L1 expression or describing microsatellite instability.

## 10. AI and Radiomics: Technical Limitations in the Current Literature

The generalizability of a predictive model depends significantly on how it is validated. In an idealized framework for model development, investigators employ a base cohort of patients for initial training and “tuning” (optimization of parameters and feature weights). Validation is then conducted using two types of datasets, which we have defined as follows: (1) a validation set (consisting of patients set apart from the original cohort and kept “unseen” by the model until the testing phase, essentially serving as an extension of the training data with similar patient demographics) and (2) a test set (a cohort that is completely independent from the training/validation sets, using patients from a separate institution or database other than the original dataset). Test sets allow for higher quality evaluation than validation sets alone, and prospective test sets provide the most rigorous method for model testing. In the 2022 review, an equal number of studies utilized cross-validation (*n* = 3, 25%), a weak form of validation that only utilizes recycled data from the training set, as either validation (*n* = 3, 25%) or test (*n* = 3, 25%) sets. Two studies did not validate their models at all, and an additional model was stress-tested with both validation and test sets. No studies used prospective, external testing cohorts.

The performances of the AI models were reported in the form of an AUC or C-index in seven studies (58.3%). Of these, four (33.3%) articles reported a model’s performance after its application to a test set, providing the most generalizable and “real-world” evaluation of accuracy. The mean performance in this group was 0.719, with the highest (0.857) observed in a small study of 50 total patients (16 in the test set) that predicted response to treatment [39]. Although this figure denotes high predictive accuracy, the very small sample size introduces doubt as to this model’s applicability. The mean performance listed above is also inflated, as two studies had model AUCs of 0.63 for treatment prediction, a value only slightly above random chance. Of the studies that did not use test tests, two reported performances on validation sets, with a mean of 0.835, and one study reported an AUC of 0.87 on a training set.

Aside from describing methods of validation, studies can be more precisely evaluated for their quality, methodological rigor, and impact on the field by using standardized scoring forms. The radiomics quality score (RQS), originally defined by Lambin et al., is a 16-component system that assigns point values to specific study characteristics such as data collection methods (prospective vs. retrospective), whether or not measures are undertaken to avoid overfitting, study validation methods, description of imaging protocols, and incorporation of biological correlates, among many others [50]. The maximum possible score is 36, and higher scores indicate more generalizable and impactful projects in AI/radiomics. Out of the 12 melanoma studies reviewed, the median [interquartile range] RQS was 12 [4], with the highest score (20) assigned to a large prospective study based on clinical trial data [45].

## 11. New Advances and Future Directions

The introduction of immunotherapy has transformed patient outcomes in oncology. Still, to provide more personalized care and reduce the risk of adverse events, novel biomarkers, including those present in medical imaging, must be developed further. Though AI and radiomics represent promising tools that may help investigators better understand individual patient response patterns, other significant efforts that have potential to impact the field must be recognized. We briefly describe a number of examples below.

### 11.1. CT-Based Sarcopenia Measurement

Sarcopenia measurement based on CT imaging is emerging as a promising biomarker for immunotherapy patient classification and monitoring. Evidence of sarcopenia at the time of diagnosis is associated with poor patient outcomes, such as decreased OS and higher complication rates, regardless of the cancer type or treatment regimen employed [23,51]. In non-small cell lung cancer (NSCLC), sarcopenia has been significantly associated with worse prognosis, disease control rate, and overall response rate [52]. In patients with advanced melanoma treated with immunotherapy, a recent meta-analysis concluded that sarcopenia was slightly associated with decreased PFS and OS and was not associated with drug toxicity [53]. However, this study was limited by its inclusion of only six relevant papers, comprising a total of 719 patients. Taking into consideration that CT imaging is already part of standard-of-care staging and routine surveillance for patients with advanced melanoma, it may prove cost-effective to quantify sarcopenia at different points in the treatment process, providing clinicians with an additional biomarker of response [54]. However, more research must be performed on large, prospective, multicenter cohorts before definitive implementation can be carried out.

### 11.2. Adaptation of Existing Imaging Techniques

Contrast-enhanced MRI, even without the extraction and analysis of radiomics features, has allowed radiologists to not only discriminate pseudoprogression from true progression, but also predict adverse events for immunotherapy-treated patients with brain metastases due to advanced melanoma [55]. Building on this, a number of recent studies showed that low absolute values for peak standard radiotracer uptake, metabolic tumor volume, and tumor lesion glycolysis in ^18^F-FDG PET imaging were associated with favorable treatment responses [56]. Higher values, indicating a tumor that is more metabolically active, are associated with tumor proliferation and overall aggressiveness. Similarly, increases in measures of glucose utilization, including baseline tumor metabolic burden as well as bone marrow and spleen metabolism, were directly correlated with worse overall outcomes [57].

### 11.3. Quantification of Inter-Lesion Heterogeneity

There is growing interest in the assessment of intra-patient inter-lesion tumor heterogeneity, a topic that is particularly relevant in the context of the dissociated responses observed with immunotherapy. A validated radiomics signature analyzing CD8+ T-cell infiltration was shown to help researchers evaluate inter-lesion heterogeneity and predict lesion response for patients with advanced melanoma treated with anti-PD-1 immunotherapy [58]. Such an approach may allow for the identification of non-responding lesions that require additional, localized treatment above and beyond systemic regimens [59].

### 11.4. Optical Coherence Tomography 

As suspicious skin lesions are typically identified visually and initially screened using the classic “ABCDE” criteria (Asymmetry, Border irregularity, Color variation, Diameter > 6 mm, and Evolution), external imaging is an understandable approach for improving melanoma evaluation. Optical coherence tomography (OCT) is one such technique, in which infrared broadband light is used to investigate and describe the architecture of superficial skin layers in real time. Because light is differentially scattered by varying cell types (including those in normal, benign, and malignant tissue), this method represents a way to non-invasively “biopsy” a lesion, thus helping to guide clinical decision making [60]. While OCT alone was found to have poor specificity for the discrimination of benign and malignant nevi, a radiomics signature developed from OCT images was shown to have a sensitivity of 98% and a specificity of 97% [61].

### 11.5. ImmunoPET Imaging

**Figure 2:**
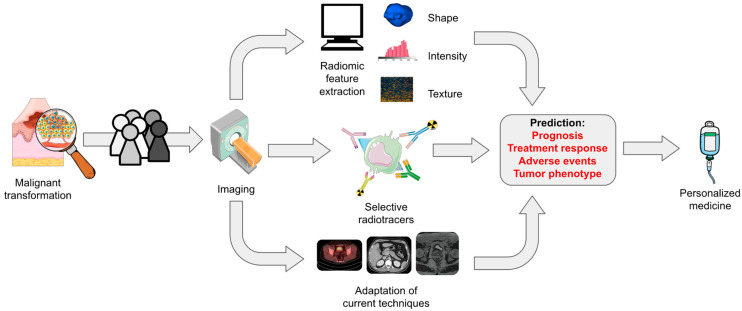
A simplified and high-level workflow example describing how radiomics and AI, selective immunoPET radiotracers, and the full utilization of current imaging technology (e.g., extracting and analyzing sarcopenia measurements, employing markers of MRI contrast enhancement, or using indicators of ^18^F-FDG-PET metabolism) could be used to create biomarkers for clinical guidance in immunotherapy-treated melanoma patients. These techniques would first be used to precisely describe a specific patient’s imaging signature. The information generated from subtle imaging biomarkers of disease could then be used to make a priori predictions about prognosis, treatment response, immune-related adverse events, and tumor phenotype in order to help clinicians decide on the best course of treatment.

The above advances notwithstanding, melanoma has historically been difficult to visualize due to superficial and mucosal invasion as well as micrometastases that elude macroscopic imaging techniques. ImmunoPET, in which receptor-specific monoclonal antibodies are conjugated with PET-avid radiotracers, is an emerging technology that could help seek out these previously hidden malignant extensions, bringing increased resolution to direct imaging by more precisely relaying the characteristics of tumor biology. Though only a few studies have been published thus far, this concept aims to directly quantify immune checkpoint inhibitor receptor expression without invasive testing [23,62]. In melanoma, a number of potential targets have been identified using murine models, including the tsMHC-II receptor (targeted by an antimouse MHC-II antibody) which typically allows tumors to evade host immune defenses [63]. In similar experimental models, tumor PD-L1 expression has been quantified using ^89^Zirconium-labeled antibody fragments [64]. The accurate quantification of tumor receptor expression can be correlated with treatment response and overall prognosis, allowing for a more refined selection of potential responders at baseline. Further still, we envision a future in which the radiomics and AI techniques described previously are used to quantify radiotracer uptake seen in immunoPET imaging, allowing for the discovery of previously unseen patterns in tracer distribution and enhancement. This theoretical combination of techniques is illustrated in Figure 2.

## 12. Conclusions

The abrupt decline in survival observed in patients diagnosed with metastatic over localized melanoma has galvanized recent research in early detection, novel therapeutics, and treatment response monitoring. Immunotherapy and AI have been leveraged together with medical imaging to approach this problem, though only a few relevant studies have thus far been published. Non-AI-based imaging biomarkers are also actively being investigated, while existing technology is being improved with the development of tumor-specific radiotracers. Irrespective of the approach employed, within the ever-evolving landscape of immunotherapy-treated melanoma, it will be critical to identify accurate and reliable non-invasive, imaging-based indicators for a priori patient selection and treatment response evaluation. There is significant promise to augment the potential of immunotherapy and, with the possible future incorporation of both clinical and genomics-based characteristics, create a holistic yet rigorously quantitative measure of classifying patients in order to guide precision therapeutics.
